# Isolation and Characterization of Microsatellite Loci in *Pistacia weinmannifolia* (Anacardiaceae)

**DOI:** 10.3390/ijms12117818

**Published:** 2011-11-11

**Authors:** Shaotian Chen, Xiayu Wu, Yunheng Ji, Junbo Yang

**Affiliations:** 1 Key Laboratory of Biodiversity and Biogeography, Kunming Institute of Botany, Chinese Academy of Sciences, Kunming 650204, China; E-Mail: chenst@kib.ac; 2 School of Life Sciences, Yunnan Normal University, Kunming 650092, China; E-Mail: xiayu98wu@yahoo.com.cn; 3 Germplasm Bank of Wild Species in Southwest China, Kunming Institute of Botany, Chinese Academy of Sciences, Kunming 650204, China

**Keywords:** *Pistacia weinmannifolia*, microsatellites, Anacardiaceae, genetic structure, polymorphism

## Abstract

Fourteen polymorphic microsatellite loci were isolated from the genomic DNA of *Pistacia weinmannifolia*, using the Fast Isolation by AFLP of Sequences Containing repeats (FIASCO) method, and screened on 12 individuals from each of two wild populations. The 14 polymorphic loci had an average of 4.1 alleles per locus varying from 1 to 9. The observed (*H*_o_) and expected (*H*_e_) heterozygosities across the two populations ranged from 0.000 to 0.933 and from 0.000 to 0.906, respectively. Tests for departure from Hardy-Weinberg equilibrium (HWE) and genotypic linkage disequilibrium (LD) were conducted for each of the two populations separately. It was found that no locus significantly deviated from HWE proportions and no significant LD was detected between loci (*p* < 0.001). In the test of cross-species utility, we successfully amplified nine (64.2%) of 14 loci in *P. chinensis* and four (28.6%) in *P. mexicana*. The relatively high level of polymorphism for these markers will facilitate further studies of gene flow, population structure and evolutionary history of *P. weinmannifolia* and its congeners.

## 1. Introduction

*Pistacia weinmannifolia* J. Poisson ex Franch (Anacardiaceae) is a shrub or arbor mainly distributed in Southwestern China including Yunnan, Sichuan, Guangxi, Guizhou and Tibet provinces, except for a few populations in Vietnam and Burma [1]. Systematically, it was a separate clade within the genus *Pistacia* supported by nuclear DNA (*ITS* and *NIA*-*i3*) and chloroplast DNA (*ndhF*, *trnC*-*trnD* and *trnL*-*F*) data [2]. Owing to its elegant profile, antibacterial properties, and capacity to repel flies and mosquitoes [3], *P. weinmannifolia* has become a popular ornamental plant, used for miniascapes, fencing and so on. Furthermore, the leaves of this plant are used in Chinese folk medicine to treat dysentery, enteritis, influenza, traumatic bleeding, headache and lung cancer [4–6].

*P. weinmannifolia* is also one of the important arid elements in its distributional region [7–9]. The species has two main kinds of habitats throughout its range: xerothemic valleys and karst regions. Because of this habitat variation, we were interested in the effects of habitat heterogeneity on gene flow and spatial genetic structure across the range of *P. weinmannifolia*. Furthermore, the habitat of this species is being lost; it was interspersed on the top of the cliffy limestone hills due to the development of modern agriculture in Guangxi, Guizhou and South Yunnan Provinces. To infer the spatial genetic pattern of the species in heterogeneous habitats and effects of habit loss or fragmentation on its genetic diversity, we need a better understanding of gene flow, population structure and evolutionary history of this species. Microsatellite markers (simple sequence repeats, SSRs) are widely used in population genetic analysis and genetic mapping due to the high variability caused by changes in their repeat numbers [10]. Hence, we describe the isolation and characterization of 14 microsatellite markers for *P. weinmannifolia*, which will facilitate our further investigations on the genetic diversity and population structure for this species.

## 2. Results and Discussion

We selected 205 positive clones to sequence with the ABI PRISM 3730XL DNA sequencer (Applied Biosystems, Foster City, CA, USA). From these, 147 sequences were found to contain SSRs of varying lengths, and 94 of them with appropriate microsatellites and sufficient flanking regions were selected to design primers. These were then screened in two wild populations. Of these loci, 14 displayed polymorphisms. Ten of these loci contained dinucleotide repeat motifs and four loci had complex repeat motifs. Details of these microsatellite loci across 24 individuals from the two wild populations are listed in Tables 1 and 2. The average allele number per locus was 4.1 (range from 1 to 9). The expected (*H*_e_) and observed (*H*_o_) heterozygosities ranged from 0.000 to 0.906, and from 0.000 to 0.933, respectively. Tests for departure from Hardy–Weinberg equilibrium (HWE) and for linkage disequilibrium (LD) were conducted for each of the two populations separately. No locus significantly deviated from HWE proportions and no significant LD was detected between loci (*p* < 0.001) in our analysis.

In the test for cross-species application of these primer pairs, nine of 14 polymorphic loci (64.2%) were successfully amplified in *P. chinensis*, and 4 (28.6%) in *P. mexicana* (Table 2).

## 3. Experimental Section

### 3.1. Isolation of Microsatellite Loci

Genomic DNA was extracted from silica-gel-dried leaves following CTAB methods and the microsatellite loci were isolated using the FIASCO protocol (Fast Isolation by AFLP of Sequences Containing Repeats) [11,12]. Approximately 500 ng of total genomic DNA was digested with *Mse*I enzyme (New England Biolabs, Beberly, MA, USA), and then fragments were ligated to the *Mse*I adaptor pair (5′-TACTCAGGACTCAT-3′/5′-GACGATGAGTCCTGAG-3′) at 37 °C for 2 h with *T*_4_ DNA ligase (Fermentas, Burlington, ON, Canada).

A diluted digestion-ligation mixture (1:10) was amplified with the adaptor-specific primers *Mse*I-N (5′-GATGAGTCCTGAGTAAN-3′), and amplified products with a size range of 200–800 bp were enriched for microsatellite repeats by magnetic bead selection with a 5′-biotinylated (AC)_15_, (AG)_15_ and (AAG)_10_ probe, respectively. Captured fragments were amplified again with adaptor-specific primers and then products were purified using an EZNA Gel Extraction Kit (Omega Bio-Tek, Guangzhou, China).

The purified PCR products with enriched microsatellite repeats were ligated into the pGEM-T vector (Promega, USA), and then transformed into DH5α cells (TaKaRa, Dalian, China). Recombinant clones were screened by blue/white selection and the positive clones were tested by PCR using (AC)_10_/(AG)_10_/(AAG)_7_ and T7/Sp6 as primers, respectively. 205 positive clones were selected to sequence with an ABI PRISM 3730XL DNA sequencer (Applied Biosystems, Foster City, CA, USA). For the microsatellites sequences containing adequate flanking regions, PCR primers were designed using the Oligo 6.0 [13].

### 3.2. Detection of Polymorphism

Polymorphisms of microsatellite loci were evaluated on 12 wild individuals of *Pistacia weinmannifolia* from each of two natural populations WX (Weixi, Yunnan: 27°40′55″N, 99°02′59″E) and XC (Xichou, Yunnan: 23°22′13″N, 104°14′25″E). Polymerase chain reactions were performed in 20 μL of reaction containing 30–50 ng genomic DNA, 0.6 μM of each primer, 7.5 μL 2× Taq PCR MasterMix [Tiangen (Tiangen, Beijing China); 0.1 U Taq Polymerase/μL, 0.5 mM dNTP each, 20 mM Tris-HCl (pH = 8.3), 100 mM KCl, 3 mM MgCl_2_], and amplifications were conducted as follows: 95 °C for 3 min followed by 30–36 cycles at 94 °C for 30 s, the optimized annealing temperature (Table 1, each primer pair was tested separately) for 30 s, 72 °C for 1 min, and a final extension step at 72 °C for 7 min. The amplified fragments were separated and visualized using the QIAxcel capillary gel electrophoresis system (QIAGEN, Irvine, CA, USA). Cross-species amplification was conducted in two closely related relative species, *P. chinensis* and *P. mexicana*, in order to test the transferability of the polymorphic primer pairs.

### 3.3. Data Analysis

Standard genetic diversity parameters of polymorphic loci were calculated using POPGENE version 1.32 [14], such as the number of alleles (*N_a_*), expected (*H_e_*) and observed heterozygosities (*H_o_*), and we also estimated deviations from Hardy-Weinberg equilibrium (HWE) and genotypic linkage disequilibrium (LD) between pairs of loci using Chi-square tests.

## 4. Conclusions

We report the developments of 14 polymorphic microsatellite markers in *Pistacia weinmannifolia*. These loci will be useful for characterizing the population genetic structure in *P. weinmannifolia* at fine and range-wide geographical scales. These loci could facilitate further studies on diversity, gene flow, mating system and phylogeography of this plant. The promising cross-taxa applicability indicated they would be potentially useful for other congeneric species of *Pistacia*.

## Figures and Tables

**Table 1:** Characteristics of 14 microsatellite loci with polymorphisms in *Pistacia weinmannifolia.*

Locus	Primer Sequence (5′–3′)	Repeat Motif	S (bp)	Ta(°C)	No.
PW001	F: 5′-AATGAGTGGAGAAGGGAAGG-3′ R: 5′-AGCAACTGTTCGCTACCCAG-3′	(AG)_13_	107	59	JN695599
PW002	F: 5′-CGAAAGATAATCAAGCTAGA-3′ R: 5′-CAACAAGGATGGACCAACAC-3′	(TC)_4_CC(TC)_5_	147	55	JN695600
PW008	F: 5′-ATCTTGAATCCTCCCACTAT-3′ R: 5′-ACAACCAAGTCACAGATAGC-3′	(AC)_9_(TC)_5_	140	52	JN695601
PW014	F: 5′-ATGCCTTTAGCAACTGAAGT-3′ R: 5′-AGTAGAGATGTATCCATGCC-3′	(AT)_7_AG(TG)_6_	178	54	JN695602
PW021	F: 5′-GCAGAAAACCAATGAAAAGC-3′ R: 5′-ACAACCAAGTCACAGATAGC-3′	(AC)_8_	206	52	JN695603
PW039	F: 5′-GCTGACTTTAGACTATTGAA-3′ R: 5′-TCATCTCTCGTTTGTGGGAC-3′	(AG)_16_	129	54	JN695605
PW047	F: 5′-AGCCTTGTGTCTGGTTTTAC-3′ R: 5′TTACAACCTTCAAACTTTAT-3′	(TC)_13_	132	49	JN695606
PW056	F: 5′-AGGTGGTAACAGTCAAGTCG-3′ R: 5′-CAGACAACCAATGAGAAGCA-3′	(AG)_16_	237	57	JN695607
PW058	F: 5′-GAAAGCCAAGCAAAGCAACA-3′ R: 5′-GGTGGAGCACAGTAACAGCA-3′	(TC)_15_	114	57	JN695609
PW060	F: 5′-CTCGAAAACCCTAATAACTT-3′ R: 5′-CATAACACCACTCACCAGGC-3′	(TG)_7_	119	55	JN695610
PW061	F: 5′-GCCACTTTTGTTCATTTCAT-3′ R: 5′-AACTCCAATTAGCTCTACAG-3′	(AG)_13_	148	50	JN695611
PW062	F: 5′-AGAGAATGAATGGGTAAAAG3′ R: 5′-CATCTTGGGTCCTCCTACTA3′	(AG)_9_	120	53	JN695612
PW081	F: 5′-GAGGGTGTGTAAGTGTTAGG3′ R: 5′-AAAAGCCACTGGTAGCACTG3′	(AG)_15_	155	52	JN695614
PW088	F: 5′-GTTACAACCAAGTCGCAGAT-3′ R: 5′-ACCTTGAATCCTCCCACTAT-3′	(AG)_14_(TG)_9_	159	57	JN695615

S: size; Ta: PCR annealing temperature; No.: GenBank Accession No.

**Table 2:** Results of initial primer screening in *Pistacia weinmannifolia.*

	WX (*N* = 12)			XC (*N* = 12)			CA

Locus	*N*_a_	*H*_e_	*H*_o_	*N*_a_	*H*_e_	*H*_o_	
PW001	2	0.464	0.083	2	0.391	0.333	−/−
PW002	4	0.663	0.25	1	0.000	0.000	c/−
PW008	4	0.663	0.333	5	0.721	0.667	c/−
PW014	2	0.290	0.000	3	0.554	0.500	−/m
PW021	3	0.540	0.250	4	0.533	0.500	−/−
PW039	2	0.228	0.250	6	0.764	0.750	−/−
PW047	3	0.353	0.250	3	0.554	0.417	c/m
PW056	5	0.739	0.833	9	0.906	0.933	−/−
PW058	5	0.757	0.833	4	0.656	0.583	c/−
PW060	3	0.518	0.417	3	0.467	0.083	c/m
PW061	2	0.4891	0.600	4	0.344	0.083	c/m
PW062	2	0.344	0.250	2	0.649	0.667	c/−
PW081	4	0.583	0.250	4	0.685	0.750	c/−
PW088	4	0.663	0.417	5	0.779	0.667	c/−

N_a_: number of alleles; H_e_: expected heterozygosity; H_o_: observed heterozygosity; WX: Population from Weixi; XC: population from Xichou; CA: cross-species application; c: successful amplification in *P. chinensis*; m: successful amplification in *P. mexicana*; -: failure in amplification.
